# Supplementary material for: DNA Hypermethylation and a Specific Methylation Spectrum on the X Chromosome in Turner Syndrome as Determined by Nanopore Sequencing
Source: J Pers Med. 2022 May 26;12(6):872. doi: 10.3390/jpm12060872 (PMC9225209; doi:10.3390/jpm12060872)
Supplement: Supplementary file 1 [file jpm-12-00872-s001.zip › jpm-1659144-supplementary.pdf]

Supplementary Materials:

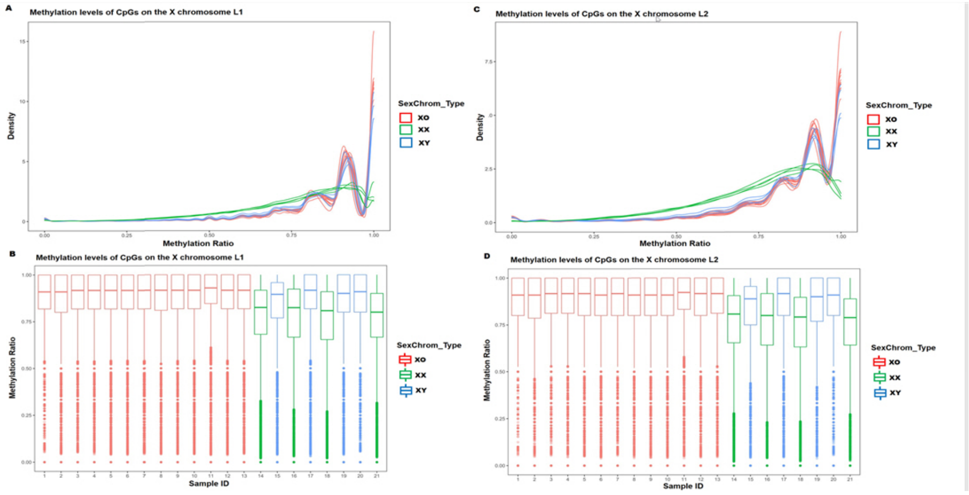

Figure S1. The methylation levels of 13 TS individuals and 8 healthy controls in the L1 and L2 regions on the chrX. The methylation density (**A**) and methylation ratio (**B**) of each sample in the L1 regions. The methylation density (**C**) and methylation ratio (**D**) of each sample in the L2 regions. (Red: TS, Green: female controls, and Blue: male controls).

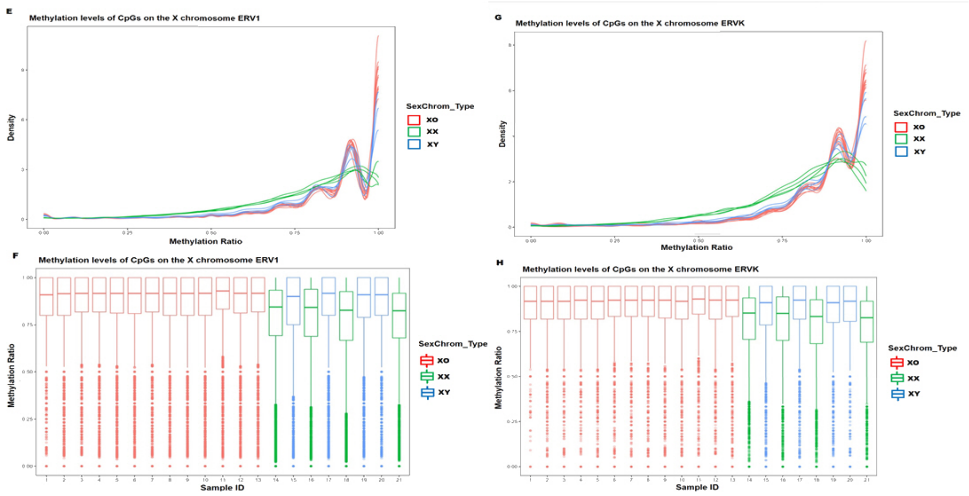

Figure S2. The methylation levels of 13 TS individuals and 8 healthy controls in the ERV1 and ERVK regions on the chrX. The methylation density (**E**) and methylation ratio (**F**) of each sample in the ERV1 regions. The methylation density (**G**) and methylation ratio (**H**) of each sample in the ERVK regions. (Red: TS, Green: female controls, and Blue: male controls).

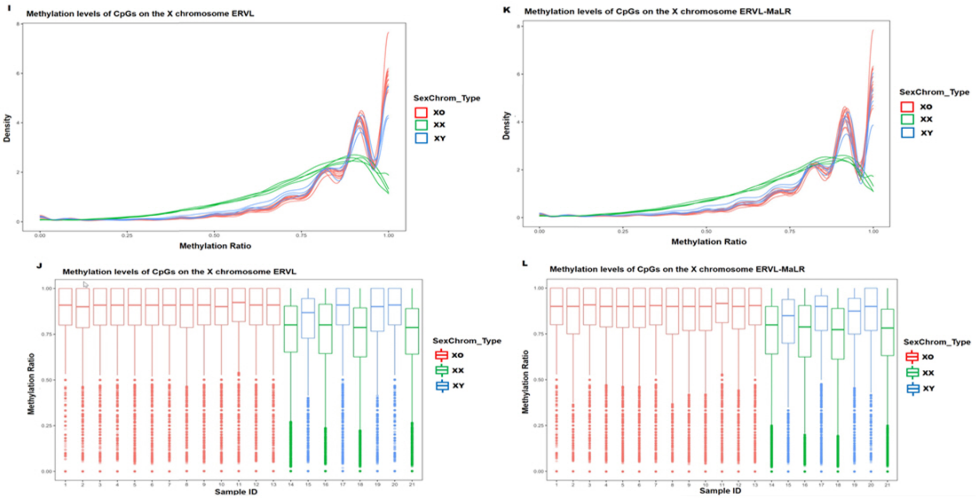

Figure S3. The methylation levels of 13 TS individuals and 8 healthy controls in the ERVL and ERVL–MaLR regions on the chrX. The methylation density (**I**) and methylation ratio (**J**) of each sample in ERVL regions. The methylation density (**K**) and methylation ratio (**L**) of each sample in the ERVL–MaLR regions. (Red: TS, Green: female controls, and Blue: male controls).

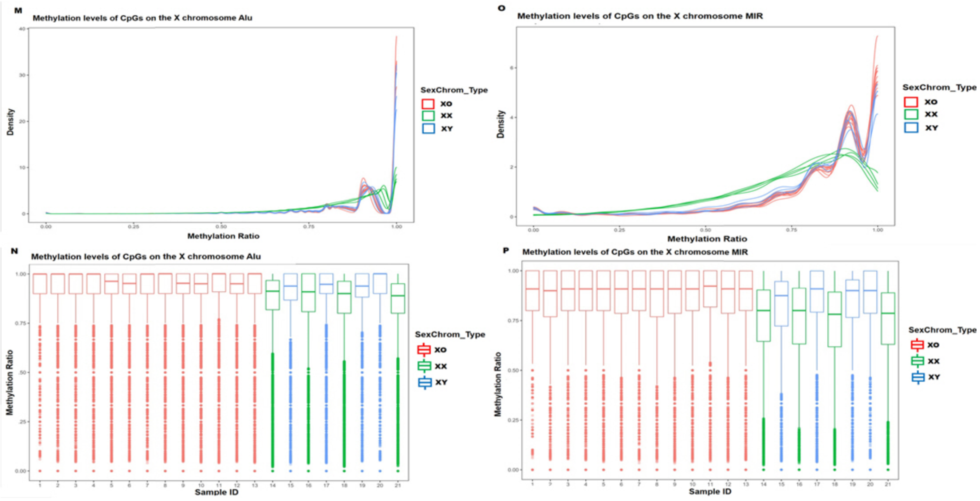

Figure S4. The methylation levels of 13 TS individuals and 8 healthy controls in the Alu and MIR regions on the chrX. The methylation density (**M**) and methylation ratio (**N**) of each sample in Alu regions. The methylation density (**O**) and methylation ratio (**P**) of each sample in the MIR regions. (Red: TS, Green: female controls, and Blue: male controls).

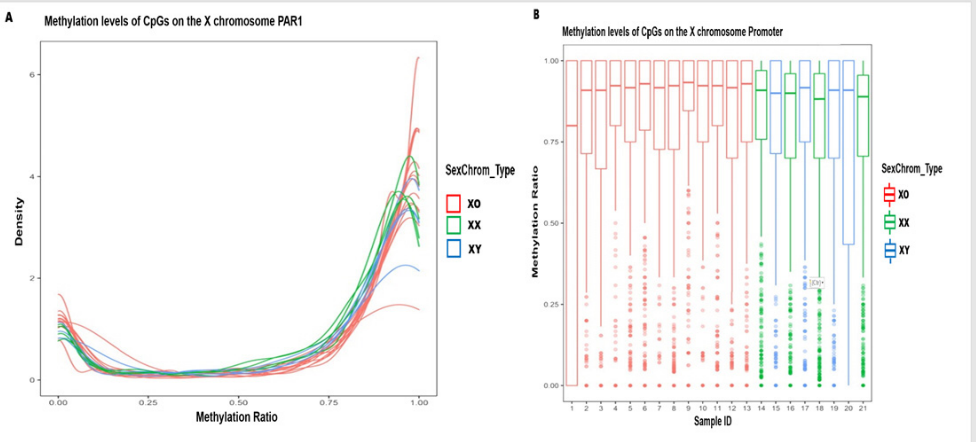

Figure S5. The methylation levels of 13 TS individuals and 8 healthy controls in the PAR1 regions on the chrX. The methylation density (**A**) and methylation ratio (**B**) of each sample in the PAR1

regions. (Red: TS, Green: female controls, and Blue: male controls).

Table S1. The phenotypes and genetic information of patients with 45X0 Turner syndrome.

**Number**	T1	T2	T3	T4	T5	T6	T7	T8	T9	T10	T11	T12	T13	Total
**Age (ys)**	8.6	11.3	9.6	10.6	12.9	3.6	9.6	9.2	9.4	11.8	3.0	6.6	3.5	
**Origin of chrX ***	P	M	P	P	M	P	M	P	P	P	P	P	M	9P/4M
**H (cm)/**	111.2	122.5	109.8	117.2	141	88.5	115.5	114.4	119.7	130	85.8	106.5	88.5	
**H-SD**	-3.7	-3.2	-4.53	-4.02	-2.55	-2.9	-3.58	-3.4	-2.5	-2.52	-2.58	-2.74	-2.87	
**W (kg)/W-SD**	26.9/	23.9/	23.2/	20.8/	47.6/	11.65/	19.5/	21.6/	23.1/	27.9/	14.1/	19.5/	12.7/	
**Phenotype**														
Short stature (HP:0004322)	+	+	+	+	+	+	+	+	+	+	+	+	+	13/13
Ptosis (HP:0000508)	-	-	-	-	-	-	-	-	-	-	+	-	-	1/13
High palate (HP:0000218)	+	+	-	-	-	+	+	-	-	-	+	-	-	5/13
Micrognathia (HP:0000347)	-	+	-	-	-	-	-	-	-	-	-	-	-	1/13
Low posterior hairline (HP:0002162)	+	+	-	-	-	-	-	-	-	-	-	-	-	2/13
Eversion of lateral third of lower eyelids (HP:0007655)	-	-	-	-	+	-	-	-	-	-	-	-	-	1/13
Prominent epicanthal folds (HP:0007930)	+	+	-	-	+	-	+	-	-	-	-	-	+	5/13
Abnormal location of ears (HP:0000357)	-	+	-	-	+	-	-	-	-	-	-	-	-	2/13
Short neck (HP:0000470)	+	+	-	-	+	-	-	-	-	-	+	-	+	5/13
Webbed neck (HP:0000465)	-	+	-	-	+	-	-	-	-	-	-	-	-	2/13
Numerous nevi (HP:0001054)	+	+	-	-	+	-	-	-	-	-	-	-	-	3/13
Shield chest (HP:0000914)	+	+	-	-	+	+	+	-	-	-	+	-	+	7/13
Wide intermamillary distance (HP:0006610)	+	+	-	-	-	+	+	-	-	-	+	-	+	6/13
Madelung deformity (HP:0003067)	+	-	+	-	-	-	-	-	-	-	-	-	-	2/13
Scoliosis (HP:0002650)	-	+	-	-	-	-	+	+	-	-	-	-	-	3/13
Multiple epiphyseal dysplasia (HP:0002654)	-	-	+	-	-	-	-	-	-	-	-	-	-	1/13
Cubitus valgus (HP:0002967)	-	+	-	-	+	-	+	-	-	-	+	-	-	4/13
Hypoplasia of the ovary (HP:0008724)	+	+	+	+	+	+	+	+	+	+	+	+	+	13/13
Hemihypertrophy (HP:0001528)	-	-	-	-	-	-	-	-	-	-	+	-	-	1/13
Hearing impairment (HP:0000365)	+	-	-	-	-	-	-	-	-	-	-	+	-	2/13
Recurrent otitis media (HP:0000403)	+	-	-	-	-	-	-	-	-	-	-	-	-	1/13

						**Cardiac malformation**								**4/13**
Bicuspid aortic valve (HP:0001647)	-	-	-	+	-	-	-	-	+	-	-	-	+	3/13
Aortic root dilation (HP:0002616)	-	+	-	+	-	-	-	-	+	-	-	-	-	3/13
						**Renal malformation**								**4/13**
Medullary nephrocalcinosis (HP:0012408)	-	-	-	-	-	-	-	-	-	+	-	-	-	1/13
Horseshoe kidney (HP:0000085)	-	-	-	-	-	-	-	-	-	-	-	+	+	2/13
Hydronephrosis (HP:0000126)	-	-	-	-	-	-	-	-	-	-	-	+	-	1/13
Nephrolithiasis (HP:0000787)	-	+	-	-	-	-	-	-	-	-	-	-	-	1/13
Specific learning disability (HP:0001328)	+	+	-	-	-	-	-	-	-	-	-	-	-	2/13
Hypothyroidism (HP:0000821)	-	+	+	-	-	-	-	-	-	-	-	-	-	2/13

* Origin of chrX: M, maternal origin and P, paternal origin.

Table S2. Differentially methylated genes identified among the three groups of Turner syndrome 45X0 and controls 46XY and 46XX.

Gene	Set	Number
CNTNAP3	XO/XXhyper	8
BCOR	XO/XXhyper	
AC004870.3	XO/XXhyper	
AK2	XO/XXhyper	
MYT1L	XO/XXhyper	
OPCML	XO/XXhyper	
GRK1	XO/XXhyper	
RP11-453F18__B.1	XO/XXhyper	
MVB12B	XO/XXhypo	117
CXorf40A	XO/XXhypo	
RPL10	XO/XXhypo	
RP4-537K23.4	XO/XXhypo	
NDUFA1	XO/XXhypo	
VMA21	XO/XXhypo	
KLF8	XO/XXhypo	
RPL36A	XO/XXhypo	
RPL36A-HNRNPH2	XO/XXhypo	
HUWE1	XO/XXhypo	
TBC1D25	XO/XXhypo	
AF196779.12	XO/XXhypo	
WDR45	XO/XXhypo	
ZNF630	XO/XXhypo	
PIM2	XO/XXhypo	
F8	XO/XXhypo	
RP11-85L21.4	XO/XXhypo	
SYP	XO/XXhypo	
TIMM17B	XO/XXhypo	
IKBKG	XO/XXhypo	
NUDT10	XO/XXhypo	
TMEM255A	XO/XXhypo	
ELK1	XO/XXhypo	
FAM127B	XO/XXhypo	
CCDC22	XO/XXhypo	
TMEM185A	XO/XXhypo	
BTBD11	XO/XXhypo	
WDR13	XO/XXhypo	
ESX1	XO/XXhypo	

PHF8	XO/XXhypo
ZNF674-AS1	XO/XXhypo
PNCK	XO/XXhypo
MAGED2	XO/XXhypo
KIAA2022	XO/XXhypo
WNK3	XO/XXhypo
G6PD	XO/XXhypo
CHST7	XO/XXhypo
ARHGEF9	XO/XXhypo
TAF9B	XO/XXhypo
TM4SF2	XO/XXhypo
TSPAN7	XO/XXhypo
EIF2AK4	XO/XXhypo
BCORL1	XO/XXhypo
PCDH19	XO/XXhypo
RP3-326L13.2	XO/XXhypo
SCML1	XO/XXhypo
CACNA1F	XO/XXhypo
WAS	XO/XXhypo
GPR50-AS1	XO/XXhypo
RP4-769N13.6	XO/XXhypo
RP4-769N13.7	XO/XXhypo
RAB33A	XO/XXhypo
SYN1	XO/XXhypo
ATP6AP1	XO/XXhypo
TRMT2B	XO/XXhypo
TRMT2B-AS1	XO/XXhypo
HS6ST2	XO/XXhypo
TIMM8A	XO/XXhypo
MORC4	XO/XXhypo
MAGIX	XO/XXhypo
IRAK1	XO/XXhypo
DLG3	XO/XXhypo
OTUD5	XO/XXhypo
AR	XO/XXhypo
AC115618.1	XO/XXhypo
HAUS7	XO/XXhypo
NHS	XO/XXhypo
MAGT1	XO/XXhypo
NUDT11	XO/XXhypo
NDUFB11	XO/XXhypo
PTCHD1	XO/XXhypo
CROCC	XO/XXhypo
U1	XO/XXhypo
BHLHB9	XO/XXhypo
HDAC6	XO/XXhypo
EBP	XO/XXhypo
LINC00086	XO/XXhypo
LONRF3	XO/XXhypo
ZBTB33	XO/XXhypo
BEX4	XO/XXhypo
ELF4	XO/XXhypo
NKAPP1	XO/XXhypo
TAZ	XO/XXhypo
RP11-66N11.8	XO/XXhypo
ZNF41	XO/XXhypo
HMGB3	XO/XXhypo
EMD	XO/XXhypo
SLC25A5-AS1	XO/XXhypo
STARD8	XO/XXhypo
MID1IP1	XO/XXhypo
MOSPD1	XO/XXhypo
FLNA	XO/XXhypo
LANCL3	XO/XXhypo
CXorf40B	XO/XXhypo
MAGEE1	XO/XXhypo

TAF7L	XO/XXhypo	
RAI2	XO/XXhypo	
SAMD11	XO/XXhypo	
SLC25A5	XO/XXhypo	
ZMYM3	XO/XXhypo	
BEX1	XO/XXhypo	
SRPX	XO/XXhypo	
IDS	XO/XXhypo	
ARHGAP6	XO/XXhypo	
UBL4A	XO/XXhypo	
RBMX2	XO/XXhypo	
PRRG1	XO/XXhypo	
PGK1	XO/XXhypo	
C1GALT1C1	XO/XXhypo	
AFF2	XO/XXhypo	
FMR1	XO/XXhypo	
LINC00629	XO/XXhypo	
SRPK3	XO/XXhypo	
IL13RA1	XO/XXhypo	
DOCK11	XO/XXhypo	
HOOK2	XO/XXhypo	
DNASE1L1	XO/XXhypo	
HOXB3	XO/XYhyper	3
HOXB-AS3	XO/XYhyper	
DTX2	XO/XYhyper	
TAF1	XO/XYhypo	
MVB12B	XO/XYhypo	
TNS1	XO/XYhypo	
AC093609.1	XO/XYhypo	16
MPL	XO/XYhypo	
EIF2AK4	XO/XYhypo	
FOXI2	XO/XYhypo	
BTBD11	XO/XYhypo	
HOXA3	XO/XYhypo	
HOXA-AS2	XO/XYhypo	
HOXA4	XO/XYhypo	
RP1-170O19.22	XO/XYhypo	
SPRN	XO/XYhypo	
CYP2E1	XO/XYhypo	
NAPRT1	XO/XYhypo	
CTU1	XO/XYhypo	
BCOR	XY/XXhyper	8
TAF1	XY/XXhyper	
RP11-453F18__B.1	XY/XXhyper	
LMTK3	XY/XXhyper	
AK2	XY/XXhyper	
SPRN	XY/XXhyper	
CYP2E1	XY/XXhyper	
AC093609.1	XY/XXhyper	
DNASE1L1	XY/XXhypo	121
FAM156B	XY/XXhypo	
DOCK11	XY/XXhypo	
AMMECR1	XY/XXhypo	
SSR4	XY/XXhypo	
FZD1	XY/XXhypo	
BEX1	XY/XXhypo	
ARX	XY/XXhypo	
EFNB1	XY/XXhypo	
C1GALT1C1	XY/XXhypo	
FAM157C	XY/XXhypo	
GPC3	XY/XXhypo	
TSPAN6	XY/XXhypo	
UBL4A	XY/XXhypo	
FMR1	XY/XXhypo	
HS6ST2	XY/XXhypo	
FAM156A	XY/XXhypo	

IDS	XY/XXhypo
AFF2	XY/XXhypo
RBMX2	XY/XXhypo
NHS	XY/XXhypo
SLITRK4	XY/XXhypo
SLC25A5	XY/XXhypo
PRRG1	XY/XXhypo
TM4SF2	XY/XXhypo
PABPC1L2A	XY/XXhypo
KIAA2022	XY/XXhypo
SRPX	XY/XXhypo
STARD8	XY/XXhypo
RAI2	XY/XXhypo
PGK1	XY/XXhypo
LANCL3	XY/XXhypo
IKBKG	XY/XXhypo
RP11-66N11.8	XY/XXhypo
FLNA	XY/XXhypo
ARHGAP6	XY/XXhypo
CXorf40B	XY/XXhypo
MOSPD1	XY/XXhypo
WNK3	XY/XXhypo
MID1IP1	XY/XXhypo
NUDT11	XY/XXhypo
HMGB3	XY/XXhypo
TAF7L	XY/XXhypo
PCDH19	XY/XXhypo
SLC25A5-AS1	XY/XXhypo
ZBTB33	XY/XXhypo
ZNF41	XY/XXhypo
RP4-537K23.4	XY/XXhypo
EMD	XY/XXhypo
SOWAHD	XY/XXhypo
LONRF3	XY/XXhypo
BCORL1	XY/XXhypo
ZMYM3	XY/XXhypo
TAZ	XY/XXhypo
EBP	XY/XXhypo
NKAPP1	XY/XXhypo
ELF4	XY/XXhypo
HDAC6	XY/XXhypo
LINC00086	XY/XXhypo
GPR50-AS1	XY/XXhypo
NDUFB11	XY/XXhypo
PTCHD1	XY/XXhypo
PHF8	XY/XXhypo
BHLHB9	XY/XXhypo
WDR45	XY/XXhypo
RPL36A-HNRNPH2	XY/XXhypo
AC115618.1	XY/XXhypo
HAUS7	XY/XXhypo
OTUD5	XY/XXhypo
MAGT1	XY/XXhypo
DLG3	XY/XXhypo
MAGIX	XY/XXhypo
IRAK1	XY/XXhypo
MORC4	XY/XXhypo
AR	XY/XXhypo
ZNF630	XY/XXhypo
TIMM8A	XY/XXhypo
RP3-326L13.2	XY/XXhypo
SYN1	XY/XXhypo
TRMT2B	XY/XXhypo
TRMT2B-AS1	XY/XXhypo
ZNF75D	XY/XXhypo
ATP6AP1	XY/XXhypo

RP4-769N13.6	XY/XXhypo
RP4-769N13.7	XY/XXhypo
WAS	XY/XXhypo
NUDT10	XY/XXhypo
CACNA1F	XY/XXhypo
RAB33A	XY/XXhypo
SCML1	XY/XXhypo
TSPAN7	XY/XXhypo
CHST7	XY/XXhypo
HUWE1	XY/XXhypo
G6PD	XY/XXhypo
ZNF674-AS1	XY/XXhypo
MAGED2	XY/XXhypo
SYP	XY/XXhypo
WDR13	XY/XXhypo
TAF9B	XY/XXhypo
PNMA6A	XY/XXhypo
ARHGEF9	XY/XXhypo
CCDC22	XY/XXhypo
FAM127B	XY/XXhypo
TMEM185A	XY/XXhypo
ESX1	XY/XXhypo
TMEM255A	XY/XXhypo
PNCK	XY/XXhypo
ELK1	XY/XXhypo
TIMM17B	XY/XXhypo
F8	XY/XXhypo
RP11-85L21.4	XY/XXhypo
PIM2	XY/XXhypo
AF196779.12	XY/XXhypo
TBC1D25	XY/XXhypo
RPL36A	XY/XXhypo
RPL10	XY/XXhypo
CXorf40A	XY/XXhypo
VMA21	XY/XXhypo
KLF8	XY/XXhypo
NDUFA1	XY/XXhypo
SRPK3	XY/XXhypo

Table S3. Enriched GO and pathway lists.

Category	Category ID	GO	Description	PARENT_GO	LogP	Enrichment	Z-score	#TotalGeneInLibrary	#GeneInGO	#GeneInHitList	#GeneInGOAndHitList	%InGO	STDV %InGO	GeneID	Hits	Log(q-value)	Gene List
GO Biological Processes	19	GO:0000423	mitophagy	19_GO:0008152 metabolic process	-5.2	33	11	30,242	28	132	4	3	1.5	292|6901|10013|10075	SLC25A5|TAFAZZIN|HDAC6|HUWE1	-0.82	MyList
GO Biological Processes	19	GO:0090042	tubulin deacetylation	19_GO:0008152 metabolic process	-4.6	53	12	30,242	13	132	3	2.3	1.3	2316|10013|56271	FLNA|HDAC6|BEX4	-0.78	MyList
GO Biological Processes	19	GO:0000422	autophagy of mitochondrion	19_GO:0008152 metabolic process	-4.5	14	7.8	30,242	81	132	5	3.8	1.7	292|6901|10013|10075|11152	SLC25A5|TAFAZZIN|HDAC6|HUWE1|WDR45	-0.78	MyList
GO Biological Processes	19	GO:0061726	mitochondrion disassembly	19_GO:0009987 cellular process	-4.5	14	7.8	30,242	81	132	5	3.8	1.7	292|6901|10013|10075|11152	SLC25A5|TAFAZZIN|HDAC6|HUWE1|WDR45	-0.78	MyList
GO Biological Processes	19	GO:0051090	regulation of DNA-binding transcription factor activity	19_GO:0050789 regulation of biological process	-3.9	4.7	5.2	30,242	440	132	9	6.8	2.2	367|2316|3654|6872|8321|8517|28952|55859|440275	AR|FLNA|IRAK1|TAF1|FZD1|IKBKG|CCDC22|BEX1|EIF2AK4	-0.25	MyList
GO Biological Processes	19	GO:1903008	organelle disassembly	19_GO:0009987 cellular process	-3.8	10	6.4	30,242	114	132	5	3.8	1.7	292|6901|10013|10075|11152	SLC25A5|TAFAZZIN|HDAC6|HUWE1|WDR45	-0.25	MyList
GO Biological Processes	19	GO:0043123	positive regulation of I-kappaB kinase/NF-kappaB signaling	19_GO:0048518 positive regulation of biological process	-3.8	7.4	5.8	30,242	186	132	6	4.5	1.8	2316|3654|7105|8517|11040|28952	FLNA|IRAK1|TSPAN6|IKBKG|PIM2|CCDC22	-0.25	MyList
GO Biological Processes	19	GO:0061912	selective autophagy	19_GO:0008152 metabolic process	-3.6	13	6.8	30,242	69	132	4	3	1.5	292|6901|10013|10075	SLC25A5|TAFAZZIN|HDAC6|HUWE1	-0.16	MyList
GO Biological Processes	19	GO:0016236	macroautophagy	19_GO:0008152 metabolic process	-3.5	5.4	5.1	30,242	295	132	7	5.3	2	292|4943|6901|8517|10013|10075|11152	SLC25A5|TBC1D25|TAFAZZIN|IKBKG|HDAC6|HUWE1|WDR45	-0.08	MyList
GO Biological Processes	19	GO:0007005	mitochondrion organization	19_GO:0009987 cellular process	-3.4	4.1	4.6	30,242	509	132	9	6.8	2.2	292|1678|4694|6901|10013|10075|10245|11152|54539	SLC25A5|TIMM8A|NDUFA1|TAFAZZIN|HDAC6|HUWE1|TIMM17B|WDR45|NDUFB11	-0.056	MyList
WikiPathways	27	WP2858	Ectoderm differentiation		-3.4	8	5.5	30,242	144	132	5	3.8	1.7	10013|23133|23229|54880|170302	HDAC6|PHF8|ARHGEF9|BCOR|ARX	-0.056	MyList

GO Biological Processes	19	GO:0046777	protein autophosphorylation	19_GO:0008152 metabolic process	-3.3	6.1	5.1	30,242	227	132	6	4.5	1.8	3654|6011|6872|11040|65267|440275	IRAK1|GRK1|TAF1|PIM2|WNK3|EIF2AK4	-0.056	MyList
GO Biological Processes	19	GO:0061919	process utilizing autophagic mechanism	19_GO:0009987 cellular process	-3.3	3.9	4.5	30,242	528	132	9	6.8	2.2	292|4943|6901|8406|8517|10013|10075|11040|11152	SLC25A5|TBC1D25|TAFAZZIN|SRPX|IKBKG|HDAC6|HUWE1|PIM2|WDR45	-0.056	MyList
GO Biological Processes	19	GO:0006914	autophagy	19_GO:0008152 metabolic process	-3.3	3.9	4.5	30,242	528	132	9	6.8	2.2	292|4943|6901|8406|8517|10013|10075|11040|11152	SLC25A5|TBC1D25|TAFAZZIN|SRPX|IKBKG|HDAC6|HUWE1|PIM2|WDR45	-0.056	MyList
Reactome Gene Sets	6	R-HSA-9609507	Protein localization		-3.1	7	5.1	30,242	163	132	5	3.8	1.7	1678|2010|6901|8266|10245	TIMM8A|EMD|TAFAZZIN|UBL4A|TIMM17B	0	MyList
GO Biological Processes	19	GO:0043122	regulation of I-kappaB kinase/NF-kappaB signaling	19_GO:0023052 signaling	-3.1	5.5	4.7	30,242	249	132	6	4.5	1.8	2316|3654|7105|8517|11040|28952	FLNA|IRAK1|TSPAN6|IKBKG|PIM2|CCDC22	0	MyList
Reactome Gene Sets	6	R-HSA-9012999	RHO GTPase cycle		-3.1	4.1	4.4	30,242	449	132	8	6.1	2.1	395|537|2010|4810|7454|9754|23229|139818	ARHGAP6|ATP6AP1|EMD|NHS|WAS|STARD8|ARHGEF9|DOCK11	0	MyList
KEGG Pathway	24	ko03022	Basal transcription factors		-3	15	6.3	30,242	45	132	3	2.3	1.3	6872|51616|54457	TAF1|TAF9B|TAF7L	0	MyList
GO Biological Processes	19	GO:0051091	positive regulation of DNA-binding transcription factor activity	19_GO:0050789 regulation of biological process	-3	5.3	4.6	30,242	260	132	6	4.5	1.8	367|3654|6872|8321|8517|55859	AR|IRAK1|TAF1|FZD1|IKBKG|BEX1	0	MyList
Reactome Gene Sets	6	R-HSA-194315	Signaling by Rho GTPases		-3	3.2	4	30,242	707	132	10	7.6	2.3	367|395|537|2010|2316|4810|7454|9754|23229|139818	AR|ARHGAP6|ATP6AP1|EMD|FLNA|NHS|WAS|STARD8|ARHGEF9|DOCK11	0	MyList
Reactome Gene Sets	6	R-HSA-76042	RNA Polymerase II Transcription Initiation And Promoter Clearance		-2.9	15	6.2	30,242	47	132	3	2.3	1.3	6872|51616|54457	TAF1|TAF9B|TAF7L	0	MyList
Reactome Gene Sets	6	R-HSA-73779	RNA Polymerase II Transcription Pre-Initiation And Promoter Opening		-2.9	15	6.2	30,242	47	132	3	2.3	1.3	6872|51616|54457	TAF1|TAF9B|TAF7L	0	MyList
Reactome Gene Sets	6	R-HSA-73776	RNA Polymerase II Promoter Escape		-2.9	15	6.2	30,242	47	132	3	2.3	1.3	6872|51616|54457	TAF1|TAF9B|TAF7L	0	MyList
Reactome Gene Sets	6	R-HSA-75953	RNA Polymerase II Transcription Initiation		-2.9	15	6.2	30,242	47	132	3	2.3	1.3	6872|51616|54457	TAF1|TAF9B|TAF7L	0	MyList

Reactome Gene Sets	6	R-HSA-167162	RNA Polymerase II HIV Promoter Escape		-2.9	15	6.2	30,242	47	132	3	2.3	1.3	6872|51616|54457	TAF1|TAF9B|TAF7L	0	MyList
Reactome Gene Sets	6	R-HSA-167161	HIV Transcription Initiation		-2.9	15	6.2	30,242	47	132	3	2.3	1.3	6872|51616|54457	TAF1|TAF9B|TAF7L	0	MyList
KEGG Pathway	24	hsa03022	Basal transcription factors		-2.9	14	6.1	30,242	48	132	3	2.3	1.3	6872|51616|54457	TAF1|TAF9B|TAF7L	0	MyList
Reactome Gene Sets	6	R-HSA-9716542	Signaling by Rho GTPases, Miro GTPases and RHOBTB3		-2.9	3.2	3.9	30,242	723	132	10	7.6	2.3	367|395|537|2010|2316|4810|7454|9754|23229|139818	AR|ARHGAP6|ATP6AP1|EMD|FLNA|NHS|WAS|STARD8|ARHGEF9|DOCK11	0	MyList
GO Biological Processes	19	GO:0043433	negative regulation of DNA-binding transcription factor activity	19_GO:0050789 regulation of biological process	-2.9	6.2	4.7	30,242	185	132	5	3.8	1.7	2316|3654|6872|28952|440275	FLNA|IRAK1|TAF1|CDC22|EIF2AK4	0	MyList
Reactome Gene Sets	6	R-HSA-1793185	Chondroitin sulfate/dermatan sulfate metabolism		--2.9	14	6	30,242	50	132	3	2.3	1.3	2719|3423|56548	GPC3|IDS|CHST7	0	MyList
GO Biological Processes	19	GO:0071478	cellular response to radiation	19_GO:0050896 response to stimulus	-2.9	6.1	4.7	30,242	187	132	5	3.8	1.7	2002|2332|6011|6872|440275	ELK1|FMR1|GRK1|TAF1|EIF2AK4	0	MyList
GO Biological Processes	19	GO:0009896	positive regulation of catabolic process	19_GO:0048518 positive regulation of biological process	-2.8	3.7	4	30,242	493	132	8	6.1	2.1	292|2332|2719|6872|8517|10075|11040|28952	SLC25A5|FMR1|GPC3|TAF1|IKBKG|HUWE1|PIM2|CCDC22	0	MyList
GO Biological Processes	19	GO:0007249	I-kappaB kinase/NF-kappaB signaling	19_GO:0023052 signaling	-2.8	4.9	4.3	30,242	281	132	6	4.5	1.8	2316|3654|7105|8517|11040|28952	FLNA|IRAK1|TSPAN6|IKBKG|PIM2|CCDC22	0	MyList
GO Biological Processes	19	GO:2000300	regulation of synaptic vesicle exocytosis	19_GO:0051179 localization	-2.8	13	5.7	30,242	54	132	3	2.3	1.3	2332|6853|6855	FMR1|SYN1|SYP	0	MyList
Reactome Gene Sets	6	R-HSA-1638091	Heparan sulfate/heparin (HS-GAG) metabolism		-2.7	12	5.7	30,242	55	132	3	2.3	1.3	2719|3423|90161	GPC3|IDS|HS6ST2	0	MyList
GO Biological Processes	19	GO:0006360	transcription by RNA polymerase I	19_GO:0008152 metabolic process	-2.7	12	5.7	30,242	55	132	3	2.3	1.3	2316|6872|23133	FLNA|TAF1|PHF8	0	MyList
Reactome Gene Sets	6	R-HSA-71387	Metabolism of carbohydrates		-2.7	4.7	4.2	30,242	295	132	6	4.5	1.8	2539|2719|3423|5230|56548|90161	G6PD|GPC3|IDS|PGK1|CHST7|HS6ST2	0	MyList

GO Biological Processes	19	GO:0010257	NADH dehydrogenase complex assembly	19_GO:0009987 cellular process	-2.7	12	5.5	30,242	57	132	3	2.3	1.3	4694|6901|54539	NDUFA1|TAFAZZIN|NDUFB11	0	MyList
GO Biological Processes	19	GO:0032981	mitochondrial respiratory chain complex I assembly	19_GO:0009987 cellular process	-2.7	12	5.5	30,242	57	132	3	2.3	1.3	4694|6901|54539	NDUFA1|TAFAZZIN|NDUFB11	0	MyList
GO Biological Processes	19	GO:0071482	cellular response to light stimulus	19_GO:0050896 response to stimulus	-2.7	7.4	4.7	30,242	124	132	4	3	1.5	2332|6011|6872|440275	FMR1|GRK1|TAF1|EIF2AK4	0	MyList
Reactome Gene Sets	6	R-HSA-1630316	Glycosaminoglycan metabolism		-2.7	7.4	4.7	30,242	124	132	4	3	1.5	2719|3423|56548|90161	GPC3|IDS|CHST7|HS6ST2	0	MyList
GO Biological Processes	19	GO:0050808	synapse organization	19_GO:0009987 cellular process	-2.6	3.8	3.8	30,242	426	132	7	5.3	2	1741|2316|6853|8321|10013|80823|139065	DLG3|FLNA|SYN1|FZD1|HDAC6|BHLHB9|SLITRK4	0	MyList
Reactome Gene Sets	6	R-HSA-1268020	Mitochondrial protein import		-2.6	11	5.2	30,242	64	132	3	2.3	1.3	1678|6901|10245	TIMM8A|TAFAZZIN|TIMM17B	0	MyList
Canonical Pathways	11	M34	PID TCR PATHWAY		-2.6	11	5.2	30,242	64	132	3	2.3	1.3	2316|7454|8517	FLNA|WAS|IKBKG	0	MyList
Reactome Gene Sets	6	R-HSA-450294	MAP kinase activation		-2.6	11	5.2	30,242	64	132	3	2.3	1.3	2002|3654|8517	ELK1|IRAK1|IKBKG	0	MyList
GO Biological Processes	19	GO:0009416	response to light stimulus	19_GO:0050896 response to stimulus	-2.5	4.3	3.9	30,242	321	132	6	4.5	1.8	778|2002|2332|6011|6872|440275	CACNA1F|ELK1|FMR1|GRK1|TAF1|EIF2AK4	0	MyList
Reactome Gene Sets	6	R-HSA-162599	Late Phase of HIV Life Cycle		-2.5	6.6	4.4	30,242	139	132	4	3	1.5	6872|51616|54457|89853	TAF1|TAF9B|TAF7L|MVB12B	0	MyList
Canonical Pathways	11	M153	PID P75 NTR PATHWAY		-2.5	10	5	30,242	68	132	3	2.3	1.3	3654|8517|55859	IRAK1|IKBKG|BEX1	0	MyList
GO Biological Processes	19	GO:0051129	negative regulation of cellular component organization	19_GO:0048519 negative regulation of biological process	-2.5	3	3.5	30,242	691	132	9	6.8	2.2	292|367|395|2316|7454|10013|10075|23133|58526	SLC25A5|AR|ARHGAP6|FLNA|WAS|HDAC6|HUWE1|PHF8|MID1IP1	0	MyList
GO Biological Processes	19	GO:0016241	regulation of macroautophagy	19_GO:0050789 regulation of biological process	-2.5	6.5	4.3	30,242	141	132	4	3	1.5	292|4943|8517|10013	SLC25A5|TBC1D25|IKBKG|HDAC6	0	MyList
GO Biological Processes	19	GO:0071214	cellular response to abiotic stimulus	19_GO:0050896 response to stimulus	-2.5	4.1	3.8	30,242	333	132	6	4.5	1.8	2002|2332|6011|6872|65267|440275	ELK1|FMR1|GRK1|TAF1|WNK3|EIF2AK4	0	MyList
GO Biological Processes	19	GO:0104004	cellular response to environmental stimulus	19_GO:0050896 response to stimulus	-2.5	4.1	3.8	30,242	333	132	6	4.5	1.8	2002|2332|6011|6872|65267|440275	ELK1|FMR1|GRK1|TAF1|WNK3|EIF2AK4	0	MyList

<table>
<tr><td>GO Biological Processes</td><td>19</td><td>GO:0045732</td><td>positive regulation of protein catabolic process</td><td>19_GO:0048518 positive regulation of biological process</td><td>-2.4</td><td>4.9</td><td>4</td><td>30,242</td><td>232</td><td>132</td><td>5</td><td>3.8</td><td>1.7</td><td>2332|2719|6872|10075|28952</td><td>FMR1|GPC3|TAF1|HUWE1|CCDC22</td><td>0</td><td>MyList</td></tr>
<tr><td>GO Biological Processes</td><td>19</td><td>GO:0022411</td><td>cellular component disassembly</td><td>19_GO:0009987 cellular process</td><td>-2.4</td><td>3.6</td><td>3.6</td><td>30,242</td><td>448</td><td>132</td><td>7</td><td>5.3</td><td>2</td><td>292|4943|6901|10013|10075|11152|58526</td><td>SLC25A5|TBC1D25|TAFAZZIN|HDAC6|HUWE1|WDR45|MID1IP1</td><td>0</td><td>MyList</td></tr>
<tr><td>Reactome Gene Sets</td><td>6</td><td>R-HSA-162906</td><td>HIV Infection</td><td></td><td>-2.4</td><td>4.9</td><td>4</td><td>30,242</td><td>234</td><td>132</td><td>5</td><td>3.8</td><td>1.7</td><td>292|6872|51616|54457|89853</td><td>SLC25A5|TAF1|TAF9B|TAF7L|MVB12B</td><td>0</td><td>MyList</td></tr>
<tr><td>Reactome Gene Sets</td><td>6</td><td>R-HSA-448424</td><td>Interleukin-17 signaling</td><td></td><td>-2.4</td><td>9.5</td><td>4.8</td><td>30,242</td><td>72</td><td>132</td><td>3</td><td>2.3</td><td>1.3</td><td>2002|3654|8517</td><td>ELK1|IRAK1|IKBKG</td><td>0</td><td>MyList</td></tr>
<tr><td>Reactome Gene Sets</td><td>6</td><td>R-HSA-167172</td><td>Transcription of the HIV genome</td><td></td><td>-2.4</td><td>9.4</td><td>4.8</td><td>30,242</td><td>73</td><td>132</td><td>3</td><td>2.3</td><td>1.3</td><td>6872|51616|54457</td><td>TAF1|TAF9B|TAF7L</td><td>0</td><td>MyList</td></tr>
<tr><td>GO Biological Processes</td><td>19</td><td>GO:0010639</td><td>negative regulation of organelle organization</td><td>19_GO:0048519 negative regulation of biological process</td><td>-2.4</td><td>4</td><td>3.7</td><td>30,242</td><td>348</td><td>132</td><td>6</td><td>4.5</td><td>1.8</td><td>292|395|7454|10013|10075|58526</td><td>SLC25A5|ARHGAP6|WAS|HDAC6|HUWE1|MID1IP1</td><td>0</td><td>MyList</td></tr>
<tr><td>Reactome Gene Sets</td><td>6</td><td>R-HSA-162587</td><td>HIV Life Cycle</td><td></td><td>-2.3</td><td>6</td><td>4.1</td><td>30,242</td><td>152</td><td>132</td><td>4</td><td>3</td><td>1.5</td><td>6872|51616|54457|89853</td><td>TAF1|TAF9B|TAF7L|MVB12B</td><td>0</td><td>MyList</td></tr>
<tr><td>GO Biological Processes</td><td>19</td><td>GO:0031346</td><td>positive regulation of cell projection organization</td><td>19_GO:0048518 positive regulation of biological process</td><td>-2.3</td><td>3.9</td><td>3.6</td><td>30,242</td><td>353</td><td>132</td><td>6</td><td>4.5</td><td>1.8</td><td>2316|2332|8321|9696|80823|139818</td><td>FLNA|FMR1|FZD1|CROCC|BHLHB9|DOCK11</td><td>0</td><td>MyList</td></tr>
<tr><td>WikiPathways</td><td>27</td><td>WP4666</td><td>Hepatitis B infection</td><td></td><td>-2.3</td><td>6</td><td>4.1</td><td>30,242</td><td>154</td><td>132</td><td>4</td><td>3</td><td>1.5</td><td>537|2002|3654|8517</td><td>ATP6AP1|ELK1|IRAK1|IKBKG</td><td>0</td><td>MyList</td></tr>
<tr><td>Reactome Gene Sets</td><td>6</td><td>R-HSA-9013148</td><td>CDC42 GTPase cycle</td><td></td><td>-2.3</td><td>5.9</td><td>4.1</td><td>30,242</td><td>155</td><td>132</td><td>4</td><td>3</td><td>1.5</td><td>7454|9754|23229|139818</td><td>WAS|STARD8|ARHGEF9|DOCK11</td><td>0</td><td>MyList</td></tr>
<tr><td>KEGG Pathway</td><td>24</td><td>hsa05230</td><td>Central carbon metabolism in cancer</td><td></td><td>-2.3</td><td>8.8</td><td>4.6</td><td>30,242</td><td>78</td><td>132</td><td>3</td><td>2.3</td><td>1.3</td><td>2539|8266|29911</td><td>G6PD|UBL4A|HOOK2</td><td>0</td><td>MyList</td></tr>
<tr><td>GO Biological Processes</td><td>19</td><td>GO:0051494</td><td>negative regulation of cytoskeleton organization</td><td>19_GO:0048519 negative regulation of biological process</td><td>-2.2</td><td>5.6</td><td>3.9</td><td>30,242</td><td>163</td><td>132</td><td>4</td><td>3</td><td>1.5</td><td>395|7454|10013|58526</td><td>ARHGAP6|WAS|HDAC6|MID1IP1</td><td>0</td><td>MyList</td></tr>
<tr><td>GO Biological Processes</td><td>19</td><td>GO:0007420</td><td>brain development</td><td>19_GO:0032502 developmental process</td><td>-2.2</td><td>2.7</td><td>3.2</td><td>30,242</td><td>753</td><td>132</td><td>9</td><td>6.8</td><td>2.2</td><td>2316|2334|2539|6872|8321|23133|57526|139411|170302</td><td>FLNA|AFF2|G6PD|TAF1|FZD1|PHF8|PCDH19|PTCHD1|ARX</td><td>0</td><td>MyList</td></tr>
</table>

Reactome Gene Sets	6	R-HSA-674695	RNA Polymerase II Pre-transcription Events		-2.2	8.2	4.4	30,242	84	132	3	2.3	1.3	6872|51616|54457	TAF1|TAF9B|TAF7L	0	MyList
Reactome Gene Sets	6	R-HSA-168142	Toll Like Receptor 10 (TLR10) Cascade		-2.2	8.1	4.3	30,242	85	132	3	2.3	1.3	2002|3654|8517	ELK1|IRAK1|IKBKG	0	MyList
Reactome Gene Sets	6	R-HSA-975871	MyD88 cascade initiated on plasma membrane		-2.2	8.1	4.3	30,242	85	132	3	2.3	1.3	2002|3654|8517	ELK1|IRAK1|IKBKG	0	MyList
Reactome Gene Sets	6	R-HSA-168176	Toll Like Receptor 5 (TLR5) Cascade		-2.2	8.1	4.3	30,242	85	132	3	2.3	1.3	2002|3654|8517	ELK1|IRAK1|IKBKG	0	MyList
GO Biological Processes	19	GO:1902904	negative regulation of supramolecular fiber organization	19_GO:0048519 negative regulation of biological process	-2.2	5.5	3.9	30,242	167	132	4	3	1.5	395|7454|10013|58526	ARHGAP6|WAS|HDAC6|MID1IP1	0	MyList
GO Biological Processes	19	GO:0046928	regulation of neurotransmitter secretion	19_GO:0051179 localization	-2.2	8	4.3	30,242	86	132	3	2.3	1.3	2332|6853|6855	FMR1|SYN1|SYP	0	MyList
GO Biological Processes	19	GO:0030901	midbrain development	19_GO:0032502 developmental process	-2.1	7.6	4.2	30,242	90	132	3	2.3	1.3	2539|6872|8321	G6PD|TAF1|FZD1	0	MyList
Reactome Gene Sets	6	R-HSA-975138	TRAF6 mediated induction of NFkB and MAP kinases upon TLR7/8 or 9 activation		-2.1	7.6	4.1	30,242	91	132	3	2.3	1.3	2002|3654|8517	ELK1|IRAK1|IKBKG	0	MyList
GO Biological Processes	19	GO:0034644	cellular response to UV	19_GO:0050896 response to stimulus	-2.1	7.6	4.1	30,242	91	132	3	2.3	1.3	2332|6872|440275	FMR1|TAF1|EIF2AK4	0	MyList
GO Biological Processes	19	GO:0042176	regulation of protein catabolic process	19_GO:0050789 regulation of biological process	-2.1	3.5	3.3	30,242	391	132	6	4.5	1.8	2316|2332|2719|6872|10075|28952	FLNA|FMR1|GPC3|TAF1|HUWE1|CCDC22	0	MyList
KEGG Pathway	24	hsa05161	Hepatitis B		-2.1	5.2	3.7	30,242	177	132	4	3	1.5	537|2002|3654|8517	ATP6AP1|ELK1|IRAK1|IKBKG	0	MyList
Reactome Gene Sets	6	R-HSA-975155	MyD88 dependent cascade initiated on endosome		-2.1	7.5	4.1	30,242	92	132	3	2.3	1.3	2002|3654|8517	ELK1|IRAK1|IKBKG	0	MyList
Reactome Gene Sets	6	R-HSA-6804756	Regulation of TP53 Activity through Phosphorylation		-2.1	7.5	4.1	30,242	92	132	3	2.3	1.3	6872|51616|54457	TAF1|TAF9B|TAF7L	0	MyList
GO Biological Processes	19	GO:0051668	localization within membrane	19_GO:0051179 localization	-2.1	2.8	3.1	30,242	651	132	8	6.1	2.1	367|1678|1741|2316|2719|8266|28952|65267	AR|TIMM8A|DLG3|FLNA|GPC3|UBL4A|CCDC22|WNK3	0	MyList

Reactome Gene Sets	6	R-HSA-168181	Toll Like Receptor 7/8 (TLR7/8) Cascade		-2.1	7.4	4.1	30,242	93	132	3	2.3	1.3	2002|3654|8517	ELK1|IRAK1|IKBKG	0	MyList
GO Biological Processes	19	GO:0033108	mitochondrial respiratory chain complex assembly	19_GO:0009987 cellular process	-2.1	7.4	4.1	30,242	93	132	3	2.3	1.3	4694|6901|54539	NDUFA1|TAFAZZIN|NDUFB11	0	MyList
GO Biological Processes	19	GO:0044089	positive regulation of cellular component biogenesis	19_GO:0048518 positive regulation of biological process	-2.1	3.1	3.2	30,242	520	132	7	5.3	2	2316|2332|7454|9696|80823|139065|139818	FLNA|FMR1|WAS|CROCC|BHLHB9|SLITRK4|DOCK11	0	MyList
GO Biological Processes	19	GO:0019693	ribose phosphate metabolic process	19_GO:0008152 metabolic process	-2.1	3.5	3.3	30,242	397	132	6	4.5	1.8	204|2539|5230|6901|55190|170685	AK2|G6PD|PGK1|TAFAZZIN|NUDT11|NUDT10	0	MyList
Reactome Gene Sets	6	R-HSA-168164	Toll Like Receptor 3 (TLR3) Cascade		-2.1	7.3	4.1	30,242	94	132	3	2.3	1.3	2002|3654|8517	ELK1|IRAK1|IKBKG	0	MyList
Reactome Gene Sets	6	R-HSA-9013423	RAC3 GTPase cycle		-2.1	7.3	4.1	30,242	94	132	3	2.3	1.3	395|2010|4810	ARHGAP6|EMD|NHS	0	MyList
GO Biological Processes	19	GO:0060322	head development	19_GO:0032502 developmental process	-2.1	2.6	3	30,242	796	132	9	6.8	2.2	2316|2334|2539|6872|8321|23133|57526|139411|170302	FLNA|AFF2|G6PD|TAF1|FZD1|PHF8|PCDH19|PTCHD1|ARX	0	MyList
GO Biological Processes	19	GO:1903827	regulation of cellular protein localization	19_GO:0051179 localization	-2.1	3	3.1	30,242	527	132	7	5.3	2	367|2010|2316|2719|9696|10075|65267	AR|EMD|FLNA|GPC3|CROCC|HUWE1|WNK3	0	MyList
Reactome Gene Sets	6	R-HSA-168138	Toll Like Receptor 9 (TLR9) Cascade		-2.1	7.2	4	30,242	96	132	3	2.3	1.3	2002|3654|8517	ELK1|IRAK1|IKBKG	0	MyList
Reactome Gene Sets	6	R-HSA-9013149	RAC1 GTPase cycle		-2	5	3.6	30,242	185	132	4	3	1.5	2010|4810|7454|139818	EMD|NHS|WAS|DOCK11	0	MyList
Reactome Gene Sets	6	R-HSA-166166	MyD88-independent TLR4 cascade		-2	6.9	3.9	30,242	99	132	3	2.3	1.3	2002|3654|8517	ELK1|IRAK1|IKBKG	0	MyList
Reactome Gene Sets	6	R-HSA-937061	TRIF(TICAM1)-mediated TLR4 signaling		-2	6.9	3.9	30,242	99	132	3	2.3	1.3	2002|3654|8517	ELK1|IRAK1|IKBKG	0	MyList
GO Biological Processes	19	GO:0060996	dendritic spine development	19_GO:0032502 developmental process	-2	6.9	3.9	30,242	99	132	3	2.3	1.3	2332|10013|80823	FMR1|HDAC6|BHLHB9	0	MyList
GO Biological Processes	19	GO:0051588	regulation of neurotransmitter transport	19_GO:0051179 localization	-2	6.9	3.9	30,242	100	132	3	2.3	1.3	2332|6853|6855	FMR1|SYN1|SYP	0	MyList

GO Biological Processes	19	GO:0006626	protein targeting to mitochondrion	19_GO:0051179 localization	-2	6.9	3.9	30,242	100	132	3	2.3	1.3	1678 | 10,075 | 10245	TIMM8A | HUWE1 | TIMM17B	0	MyList
GO Biological Processes	19	GO:0050890	cognition	19_GO:0032501 multicellular organismal process	-2	3.9	3.3	30,242	296	132	5	3.8	1.7	2334 | 80823 | 84061 | 139411 | 440275	AFF2 | BHLHB9 | MAGT1 | PTCHD1 | EIF2AK4	0	MyList
Reactome Gene Sets	6	R-HSA-9633012	Response of EIF2AK4 (GCN2) to amino acid deficiency		-2	6.8	3.9	30,242	101	132	3	2.3	1.3	6134 | 6173 | 44,0275	RPL10 | RPL36A | EIF2AK4	0	MyList
Reactome Gene Sets	6	R-HSA-168188	Toll Like Receptor TLR6:TLR2 Cascade		-2	6.8	3.9	30,242	101	132	3	2.3	1.3	2002 | 3654 | 8517	ELK1 | IRAK1 | IKBKG	0	MyList
Reactome Gene Sets	6	R-HSA-166058	MyD88:MAL(TIRAP) cascade initiated on plasma membrane		-2	6.8	3.9	30,242	101	132	3	2.3	1.3	2002 | 3654 | 8517	ELK1 | IRAK1 | IKBKG	0	MyList
GO Biological Processes	19	GO:0050821	protein stabilization	19_GO:0065007 biological regulation	-2	4.8	3.5	30,242	191	132	4	3	1.5	2316 | 6872 | 11040 | 51616	FLNA | TAF1 | PIM2 | TAF9B	0	MyList

Table S4. Candidate genes of X0_Hypomethylated which were also detected as hypomethylated in TSvs46XX in previous research.

PNAS2020	CLINEPI2015	X0hypo	PNAS2020∣CLINEPI2015	PNAS2020∣X0hypo	CLINEPI2015∣X0hypo	PNAS2020∣CLINEPI2015∣X0hypo
RP11-1148L6.9	38961	MVB12B	RBM3	NUDT10	CYP2E1	WDR45
PFKFB1	40057	RP4-537K23.4	FTSJ1	TAF9B	HOXA4	CXorf40B
XX-FW83563B9.5	TLE1	AF196779.12	EFNB1	TIMM17B	EMD	NDUFA1
SNORA70	MGC3207	ZNF630	IGBP1	ZNF674-AS1	CXorf40A	NDUFB11
AL049564.1	FAM83A	RP11-85L21.4	APEX2	FAM127B	SYP	ESX1
LINC00894	GLUD1	IKBKG	CSTF2	RP4-769N13.6	OTUD5	IDS
AIFM1	CST9L	BTBD11	RNF113A	TRMT2B	UBL4A	CHST7
AF196970.3	CCL26	TM4SF2	PQBP1	PCDH19	WNK3	PIM2
FAM127A	NPR2	EIF2AK4	LDOC1	RAI2	ZMYM3	C1GALT1C1
SLC25A53	HLA-DQB2	CACNA1F	RBM10	NKAPP1	AFF2	TAF1
ERCC6L	SEC31L2	GPR50-AS1	SLC25A14	RPL36A-HNRNPH2	ARHGEF9	SYN1
FAM104B	SH3BP2	TRMT2B-AS1	NKAP	F8	MOSPD1	ELK1
HNRNPH2	LYPD3	AC115618.1	ATG4A	TMEM255A	BCORL1	G6PD
OGT	ZNF593	HAUS7	PSMD10	NUDT11	RBMX2	LONRF3
FANCB	NAV1	CROCC	DKC1	VMA21	NHS	RPL36A
ZNF674	HP	U1	DACH2	RP11-66N11.8	PNCK	MAGT1
OOEP	DHODH	LINC00086	SOX3	HUWE1	PGK1	KLF8
RLIM	AIF1	TAF7L	YIPF6	HS6ST2	TSPAN7	CCDC22
DPPA5	STMN1	SAMD11	PRAF2	RP3-326L13.2	TAZ	ARHGAP6
ZNF81	CIAS1	DOCK11	GPR101	MORC4		WAS
AL022578.1	ZFYVE19	HOOK2	GLA	TBC1D25		MAGEE1
RP11-308D16.2	GPR109B	TNS1	UTP14A	LINC00629		HDAC6
GPKOW	FYB	MPL	MOSPD2	KIAA2022		TIMM8A
RP13-228J13.9	VAMP5	FOXI2	DDX43	TMEM185A		FLNA
PDZD11	C20orf75	HOXA3	TFE3	AC093609.1		BHLHB9
RP11-348F1.2	TACSTD1	HOXA-AS2	ARMCX5	BEX4		SCML1
RP11-348F1.3	GPR109A	RP1-170O19.22	RPL39	MAGIX		ZBTB33
SPACA5	HTRA4	SPRN	KIF4A	PRRG1		EBP
ZNF182	AMT	NAPRT1	ATRX	PTCHD1		SRPX
THOC2	P2RY2	CTU1	TSPYL2	SLC25A5-AS1		ZNF41
CLCN5	VENTX		MID2	RP4-769N13.7		HMGB3
STK26	FLJ30046		ZCCHC12	SRPK3		IRAK1
TAB3-AS2	F12		NR0B1			IL13RA1
SMIM10	STK31		ZNF449			LANCL3
KRBOX4	ZNF541		OPHN1			AR
ZNF75D	C14orf162		SLC35A2			DLG3
PIH1D3	TRPM2		MAGEH1			DNASE1L1
NUP62CL	DEFA1		IDH3G			PHF8
AL590410.1	EPN3		RBMX			RAB33A
BTK	CCIN		PIGA			SLC25A5
MAMLD1	MAN2A2		COX7B			ELF4
FAM127C	LMO2		ARMCX3			WDR13
SNORD61	CLEC4C		FUNDC2			MAGED2
SMIM10L2B	EPAS1		RAB39B			MID1IP1
AC234775.1	ARS2		NSDHL			RPL10
ARMCX3-AS1	BRD4		CETN2			STARD8
ZCCHC18	C6orf68		NKRF			FMR1
NCL	DYRK4		WDR44			ATP6AP1
HSPB1P2	HPS4		SUV39H1			BEX1
H3F3AP1	TMEM102		ZDHHC15			
RP1-161N10.1	LTBR		LAS1L			
SMAD5-AS1_2	POR		MSN			
RHOXF1P1	ZNF124		FHL1			
CXorf57	RABGEF1		ACSL4			
ZNF280C	AMDHD1		ARAF			
CELSR3-AS1	PPP4R2		MCTS1			
OVCH1-AS1	HMGN1		HDAC8			
AMER1	CIDEB		CXorf56			
DGKK	NFE2		PRDX4			

RAC1P4	NR1I2	ABCB7
ARHGAP36	SFTPD	SLITRK4
LINC01201	HSPA2	KLHL34
GPRASP1	BRCA2	RRAGB
RP11-314A15.2	HAL	XKRX
FAM122B	SLC22A3	XK
FGF13-AS1	FBXL5	MECP2
AC131011.1	ITGB1BP1	PGRMC1
MBNL3	RPS6KC1	IL1RAPL2
HDX	ENTPD1	FAM58A
PBDC1	ARID3A	PHKA1
APOOL	APOB	CNKSR2
TSR2	P2RY13	MBTPS2
RP1-274L7.1	LPO	PCSK1N
RP6-24A23.3	BTBD6	HTATSF1
MYCLP1	SERPINF1	FAM122C
TSPAN6	ZNF266	NONO
RP3-389A20.5	CTBP1	FGD1
LAGE3	BIRC4BP	RAP2C
SAT1	KLF1	POU3F4
RP13-314C10.5	RAB8A	SNX12
FAM156A	CD244	SH3BGRL
ZC4H2	HSPC159	SMS
HMGN5	TIGD5	FAM50A
KANTR	LGP1	UPF3B
RP11-236P24.3	IL27	INGX
PRICKLE3	CACYBP	CHM
NXT2	CTSG	SYTL4
MORF4L2-AS1	RPS6KA1	PIN4
LINC00893	FLJ25084	ATP6AP2
RP1-137H15.2	IFITM3	ARX
PCYT1B-AS1	SCMH1	UXT
LINC01285	GPR21	PHKA2
SMIM10L2A	AQP9	GNL3L
WDR53	LYAR	MTM1
ZXDB	PRTN3	GABRQ
AL158069.1	FLJ40919	MTMR8
FAM155B	GSG1	TMLHE
Y_RNA	LYZ	AMOT
snoU13	H2BFS	NAP1L2
MID1IP1-AS1	SEPT10	ZIC3
CENPI	ELA2	SLITRK2
LINC01381	UNQ830	GPC4
UXT-AS1	CEACAM6	MAGEE2
TLE1P1	NKG7	ATP7A
RNU6-800P	IER2	UBE2A
RP13-43E11.1	SPAG4	PRPS2
AL035088.1	SGK2	EDA
LINC01560	LAIR1	TCEAL1
RPS26P11	SLC44A2	PRPS1
VPS28	PABPN1	MTMR1
MSL3	PARVG	USP51
ZDHHC9	ACYP2	CDX4
MIR718	CLEC5A	TCEAL8
RP11-234P3.2	BTN1A1	RPGR
RP5-1091N2.9	KLHDC7B	GSPT2
C17orf98	GALNT3	LAMP2
DDX26B-AS1	GPR97	HIST1H3E
RP13-210D15.8	RBM35B	GLUD2
BTF3P8	KCNE1	RP2
RAP2C-AS1	GATAD2B	RPS6KA3
CDR1-AS	C18orf22	PAK3
BX842568.4	HIST1H2AJ	TCEAL2
RPS6KA6	ATP6V1E2	GPC3
CXorf58	KCNQ1	SLC10A3
APOO	FUT7	STAG2

MAP3K15	CYP27B1	GRIA3
ENOX2	LOC158160	TMSB4X
SASH3	FCGR3B	MAOA
MIR5003	P518	PDK3
KIF13A	CLEC9A	TBX22
FCF1P4	CSF3R	ARMCX6
PJA1	KNS2	HPRT1
RP11-287D1.4	KIAA0427	DYNLT3
NRK	GNS	SLC9A6
RP5-1000K24.2	GLRX2	SLC7A3
CFP	C6orf105	SCML2
NCBP2L	LGALS4	USP11
FGF13	CLEC12A	ARMCX1
DDX26B	MGC26963	TMEM47
DUSP9	CCT6A	IL1RAPL1
RP13-188A5.1	GPR84	ZNF185
CENPVP3	ZNF696	ABCD1
BRCC3	SLPI	SLC16A2
CMC4	MGC11257	UBQLN2
GRIPAP1	FLJ33860	PORCN
LINC01278	TSPAN16	ARHGAP4
APLN	H2AFY	ZXDA
UPRT	FLJ35773	PHF6
AL772400.1	LOC92345	ERAS
DERL2	C14orf58	PLP2
POLA1	CIZ1	PDHA1
XIAP	BRE	CLCN4
POU5F1	SLC22A18	
AC234031.1	PILRA	
GPR50	ECRG4	
BX842568.1	LCE3A	
RP11-641J8.1	FLJ35530	
ZNF711	LRG1	
AC003001.1	FKBP11	
SLC25A43	SCRL	
RNF128	RNF14	
RP11-521C20.5	FXYD1	
SOCS5P4	LYCAT	
FOXO4	SLC45A1	
AC005041.11	PNPLA2	
CTD-2269E23.4	CSTA	
RP1-164F3.8	MYL9	
TUBGCP3	HIPK3	
AL691477.1	TATDN1	
TREX2	IL21R	
RNU6-894P	PREX1	
RP11-21G20.3	SH3BP5	
RP1-169K13.3	ANKMY1	
KLHL13	DOPEY2	
AMMECR1-IT1	LOC132321	
RNU6-481P	VNN3	
CITED1	AGXT	
MIR3202-2	C15orf5	
RP5-972B16.2	MGC41945	
BX842568.2	APOA4	
NHSL2	CENPH	
GPR45	P2RX1	
GHc-857G6.7	PIP3-E	
MIR106A	SMPD3	
ZNF157	TETRAN	
CHD4	LRP8	
WBP5	C1orf150	
RP11-68I3.4	CLTCL1	
CCDC160	H1F0	
CHIC1	SEC61G	
MAP2K4P1	GPR25	

RBM41	HK2
TMEM263	DEFB1
RP11-412D9.4	NDUFS2
LINC00630	ART3
RP4-613B23.1	CLCA2
LINGO3	CYP7B1
RP11-149B9.2	GRB7
SPIN3	MS4A6A
COL4A5	DLK1
RP11-62J1.4	IFNGR2
RP11-548B3.3	FOLR3
SOWAHD	FCN1
FZD2	LCN2
RP11-320G24.1	XYLB
AF196972.9	IL19
FMR1-AS1	URP2
JADE3	ZNF710
OR11N1P	RPUSD3
RP1-149M18.4	THSD3
CFAP47	FFAR3
FMR1-AS1_2	KCNQ1DN
RNF5P1	SPATC1
PRDM9	EIF2B3
PNMA5	SYCP1
CH17-340M24.3	ABTB1
BEX5	RNASE3
TMSB15A	PTGFRN
ARHGEF6	FUT3
OSBPL5	NOL3
RP5-998H6.2	SLC26A8
CSNK1G2P1	USP10
MYCBPAP	NAGPA
RP11-94C24.6	TRIM50C
	CALML4
	TIGD2
	SERPINB10
	C3orf18
	SPI1
	GABRR1
	EPHA2
	TFAP2E
	ACPP
	LEP
	SLC37A1
	LOC201164
	CEBPE
	GP5
	PRR3
	AZU1
	TRIP13
	BSN
	KCNA6
	SLC22A12
	K6IRS2
	C3orf35
	RAB34
	TP53I3
	BCL2
	TREM2
	TACSTD2
	MPO
	CEP1
	CSPG5
	CASP6
	C19orf24

CPM
PSMD5
DNAH3
MST1
VWCE
RAC3
ALOX12
C7orf29
VTN
GNPDA1
HIVEP3
LYK5
1-Mar
CAMP
NPFFR2
CEACAM3
SLC19A1
LRPAP1
SERGEF
UCK1
FCGRT
CBX7
PC
STARD6
C9orf142
BXDC1
EMILIN1
PLCB2
PPT2
CD1D
DYDC1
LOC339768
ZNF22
SLC11A1
C17orf44
EGFL7
DDAH1
RPL31
MLX
PWP1
RTEL1
EDG3
RETN
C21orf13
LOC284948
CD59
ZFP41
MLSTD1
ZPBP2
CTHRC1
C20orf135
EIF4E
FSD1NL
ELOVL3
EPHA3
PPEF2
TXNRD2
S100P
MRPS18C
AVIL
CRISPLD2
SNTB1
CDSN
PDE3B
RALGPS2
CBFA2T3

RNPS1
FCGR2A
S100A9
B4GALT2
CD22
KCTD12
C8orf45
FLJ21820
LAIR2
CTAGE5
C19orf33
MGC39715
KIF12
ZNF169
TESSP1
MAP3K14
SLC15A4
MRGPRX3
DOK3
MERTK
KCNK10
LOC284837
LLGL2
SRD5A1
PPP2R4
FGFR1
SERPINA1
PRSSL1
RNF126
KCNA3
P2RY12
NCOR2
ELMO3
SNN
PHB2
SIGLEC5
CNGB1
AVP
TIMM13
INPP5D
GLT1D1
GPLD1
AK1
RPS2
ASGR2
TNFRSF9
GNE
SLC7A8
SULT1C2
C8orf44
F2RL2
BHMT2
C16orf50
FSTL3
ZNF553
NNAT
SLC43A3
FLJ46072
UHMK1
RSHL1
SYNE2
JAK2
LR8
NDUFAF1
SLC39A14
C10orf93

SOD3
TSPYL5
HIST1H4L
ARMC2
KBTBD5
TMEPAI
MARCO
FAM83F
TAAR5
FOXL1
TNFSF13B
PLXDC1
ZNF454
C6orf139
HRASLS5
TRIM16
TRIP6
PODXL2
GPR77
MGMT
PTPRJ
MRPL28
POU3F1
TRPM6
HSPBP1
UNQ3033
HNRPF
SEMA3B
MPST
CDH5
ANKRD13C
SLC23A1
SUMO3
TP53TG3
SIGLEC9
TYROBP
TMSL8
BCAP31
DXS9879E
F8A1
SLC9A7
OCRL
RP1-112K5.2
MPP1
VBP1
ARMCX4
CXorf34
REPS2
GK
PDZD4
MGC39606
PLXNA3
NGFRAP1
FAM70A
POLA
MED12
PHF16
MAGED4
FLJ20298
MTCP1
TSC22D3
CD99L2
FAM11A
RP11-450P7.3
CXX1
BEX2

GLT28D1
CASK
AMMECR1
ZNF673
FLJ10178
CXorf9
FAM3A
MASK
CXorf26
IGSF1
RP11-311P8.3
ARMCX2
SH3KBP1
MSL3L1
IRS4
GABRE
TMEM32
BIRC4
TRO
CXorf17
HADH2
CXorf42
FLJ14503
PDCD8
CDKL5
DKFZp564K142
BRWD3
RNF12
FLJ30058
XPNPEP2
FLJ25444
WWC3
LOC389852
LMO6
FLJ39827
TMEM29
TMEM164
CXorf53
CHRDL1
MLLT7
ARD1A
RP3-473B4.1
RAB9B
TLR8
GPRASP2
CXorf38
CXorf41
SSR4
GABRA3
PFC
PCYT1B
GPR173
FLJ11016
DRP2
ITM2A
MYCL2
SMARCA1
LOC203427
SUHW3
STK23
EGFL6
ZNF6
FLJ31204
ITGB1BP2
CLDN2
AVPR2

BMX
FSHPRH1
KIAA1166
ZCCHC5
NSBP1
GAB3
COVA1
NOX1
RBBP7
MORF4L2
FRMPD4
MAGED1
PNPLA4
L1CAM
CXorf21
TCEAL3
TMEM35
TCEAL4
CYSLTR1
NLGN3
PCTK1
CLIC2
GLRA2
PLXNB3
AGTR2
TLR7
BGN
RENBP
PRRG3
SMC1L1
KCND1
CXorf12
